# Survival Benefits of Anti-PD-1 Therapy in Combination With Radiotherapy in Chinese Melanoma Patients With Brain Metastasis

**DOI:** 10.3389/fonc.2021.646328

**Published:** 2021-03-18

**Authors:** Shuang Wu, Chuanping Yuan, Lei Chen, Lanlan Guo, Yong Chen, Zhenwei Peng, Lixia Lu

**Affiliations:** ^1^Department of Radiation Oncology, The First Affiliated Hospital of Sun Yat-sen University, Guangzhou, China; ^2^Department of Oncology, Xinyu People's Hospital, Xinyu, China; ^3^State Key Laboratory of Oncology in South China, Guangdong Key Laboratory of Nasopharyngeal Carcinoma Diagnosis and Therapy, Collaborative Innovation Center for Cancer Medicine, Sun Yat-sen University Cancer Center, Guangzhou, China; ^4^Department of Radiation Oncology, Sun Yat-sen University Cancer Center, Guangzhou, China; ^5^Clinical Trials Unit, The First Affiliated Hospital of Sun Yat-sen University, Guangzhou, China; ^6^Institute of Precision Medicine, The First Affiliated Hospital of Sun Yat-sen University, Guangzhou, China

**Keywords:** melanoma, anti-PD-1, radiotheapy, brain metastasis, immunotherapy

## Abstract

Limited data reported the synergistic anti-tumor effect of anti-PD-1 (programmed death 1) therapy and radiotherapy on melanoma BM (brain metastasis). And the efficacy in the Chinese population is unclear. This study aimed to evaluate the efficacy of anti-PD-1 therapy and radiotherapy in Chinese melanoma patients with BM. We retrospectively reviewed 96 consecutive melanoma patients with BM treated at Sun Yat-Sen University Cancer Center. Patient demographics, BM characteristics and treatment details were carefully collected. The intracranial PFS (progression free survival) and OS (overall survival) were estimated using the Kaplan-Meier method. Twenty-five patients were treated with anti-PD-1 therapy and radiotherapy. Eighteen (72.0%) patients had SBRT (stereotactic body radiation therapy) or SRS (stereotactic radiosurgery) for BM, 1 (4.0%) patient had WBRT (whole brain radiation therapy), 6 (24.0%) patients had SBRT/SRS and WBRT. The median treatment period of anti-PD-1 therapy was 10.77 months. Objective intracranial response was observed in 15 (60%) patients, and 5 (20%) patients achieved CR (complete response). After a median follow-up of 16 months, 11 (44%) patients experienced intracranial PD (progressive disease), and 15 (60%) patients died. The median intracranial PFS and OS were 10.73 months (range, 1.67–38.83 months) and 15.87 months (range, 2.47–41.50 months), respectively. The 1-year intracranial PFS and OS were 61.9% (95% CI, 44.1–86.9%) and 62.5% (95%CI, 45.8–85.2%), respectively. Patients with BM can benefit from a combination of anti-PD-1 therapy and radiotherapy. It merits further investigation in melanoma patients with BM.

## Introduction

Malignant melanoma is a commonly reported type of skin cancer in Western countries (1, 2). And it also poses an increasing threat to the health of the Chinese population. Between 1990 and 2017, the annual incidence and prevalence rate of melanoma in China increased significantly, far beyond the global level (3). The clinical and biological characteristics of melanoma differ greatly between Caucasian and Chinese patients (4, 5). Instead of cutaneous melanomas as the major subtype in Caucasian patients, ~70% of Chinese patients are diagnosed with acral (42.8%) or mucosal melanoma (27.0%) (4, 6). It is generally believed that patients with acral and mucosal melanoma portend a worse prognosis (7). Therefore, it is essential to explore effective treatments for the Chinese population.

Melanoma is the third most common malignant tumor to metastasize to the brain, after lung, and breast cancer. The reported incidence of BM (brain metastasis) in patients with melanoma was 10–40%, even higher (>70%) in the autopsy series (8–10). The prognosis of patients with BM is extremely poor. The median OS (overall survival) from diagnosis of BM was only 4–6 months in unselected patients (10–13). The survival of patients with BM undergoing surgery and/or radiotherapy only slightly improved, with a median OS of 6.4–10.8 months (10, 12–14). Although targeted therapy using BRAF/MEK inhibitors has dramatically changed the prognosis of patients with metastatic melanoma (15), patients with BM who received targeted therapy did not have a significant improvement in survival, with a median OS of 7–9.6 months (16, 17).

More recently, immunotherapy with anti-PD-1 (programmed death-1) antibodies has shown impressive and durable responses in patients with melanoma (18–22). However, the benefit of anti-PD-1 therapy combined with radiotherapy in melanoma patients with BM remains unclear, especially in the Chinese population. Therefore, in the present study, we reported the efficacy of this combined treatment strategy in the Chinese population.

## Patients and Methods

### Patients

**Figure 1:**
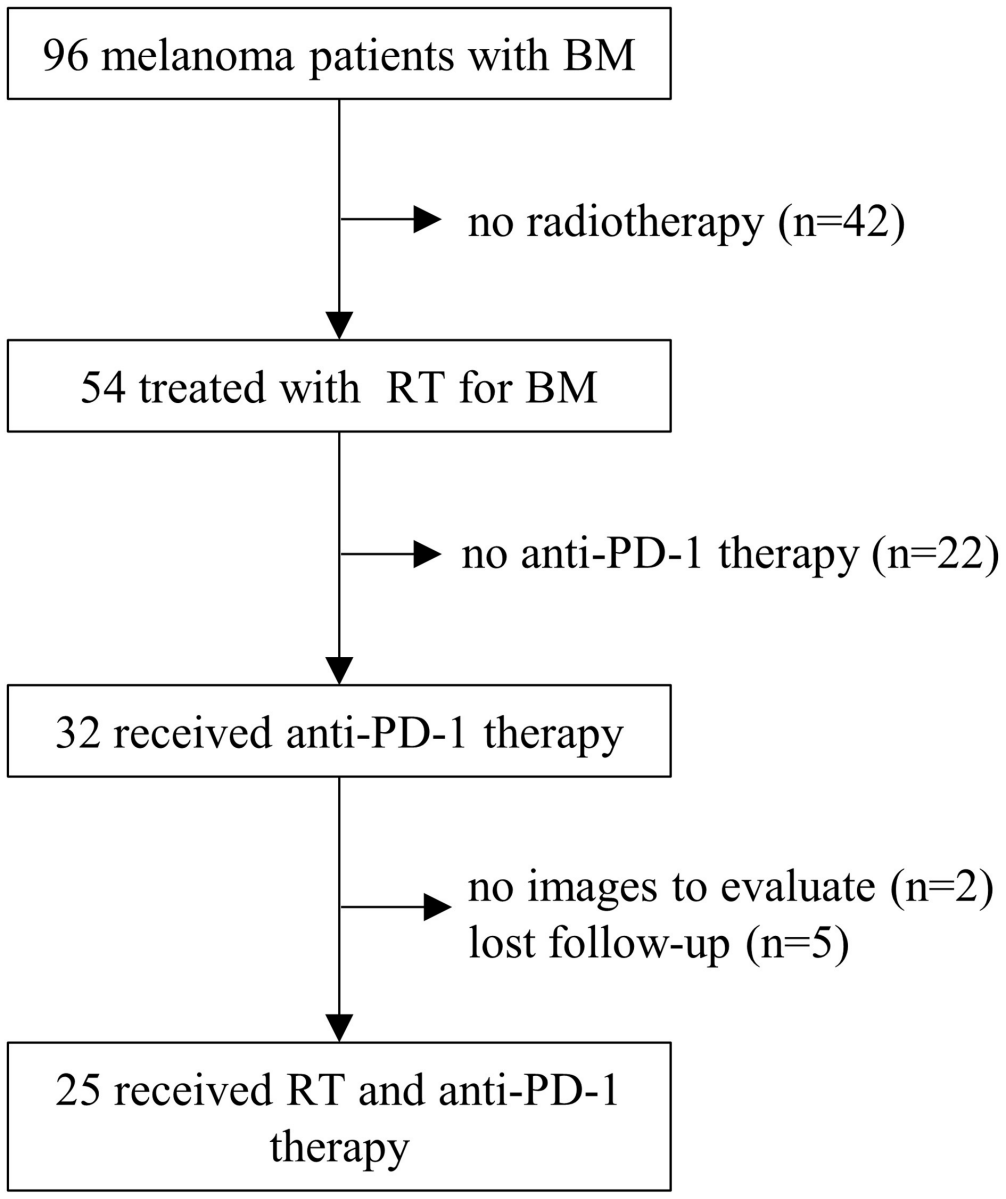
Patient screening. BM, brain metastasis; RT, radiotherapy; PD-1, programmed death 1.

This retrospective study was approved by our institutional review board, and the requirement to obtain informed consent was waived. Between August 2010 and September 2019, a total of 96 consecutive melanoma patients with BM were treated at Sun Yat-Sen University Cancer Center. Patients who met the following criteria were enrolled for analysis: (1). histologically confirmed melanoma; (2). BM confirmed by MRI or CT scan; (3). KPS (Karnofsky Performance Status) ≥70; (4). received ≥1 dose of anti-PD-1 therapy; (5). received ≥1 course of radiotherapy for BM; (6). at least one follow-up MRI or CT examination after treatment. Patients who had no baseline images were excluded. Finally, 25 melanoma patients with BM were included in the present study (Figure 1).

### Data Collection

The following data were collected for each patient: baseline demographics, KPS score, initial diagnosis date of melanoma, gene mutational status (BRAF/c-Kit), serum LDH (lactate dehydrogenase) level, stage of disease; initial diagnosis date of BM, the baseline number of BM lesions, the maximum diameter of BM, symptoms of BM, steroids use, number of extracranial metastasis sites; details of anti-PD-1 therapy and radiotherapy, prior systemic treatments, prior local therapy to BMs; time to endpoint data. Endpoints evaluated were intracranial PFS (progression-free survival) and OS.

### Statistics

The follow-up period was counted from the date of diagnosis of BM, which was defined as the first radiological diagnosis date of BM. The treatment efficacy was assessed according to the Response Evaluation Criteria in Solid Tumors (RECIST) version 1.1 (23). The intracranial PFS and OS rates were evaluated using the Kaplan–Meier method. The intracranial PFS was calculated as the time from diagnosis of BM to progression of the intracranial lesions or death due to any cause. The OS was defined as the time from diagnosis of BM to death due to any cause. The melanoma-molGPA score (24) was calculated based on age, KPS score, number of extracranial metastasis sites, number of BMs, and BRAF mutation status for each patient. The association between melanoma molGPA score and the estimated intracranial PFS or OS was tested using the log-rank test. The treatment related toxicities were evaluated according to CTCAE (Common Terminology Criteria for Adverse Events) v4.03. A two-sided *P* ≤ 0.05 was considered statistically significant. All the analyses were performed using R (version 3.6.0).

## Results

### Patient Demographics

Between August 2010 and September 2019, a total of 25 melanoma patients with BM who received anti-PD-1 therapy and radiotherapy were identified (Figure 1). The median follow-up after diagnosis of BM was 16 months (range, 2.5–41.5 months). The baseline patient characteristics are shown in Table 1. The median age of patients was 48 years old (range, 25–77 years). Most patients were female (52.0%). All patients were in good conditions with a KPS score ≥70. Seventeen (68.0%) patients were diagnosed with acral or mucosal melanoma, and 8 (32.0%) patients were diagnosed with cutaneous melanoma. Eleven patients (44.0%) were positive for BRAF V600 mutation. C-kit mutations were detected in only two patients (8.0%). At the time of diagnosis of BM, only 3 patients (12.0%) had an elevated LDH level. The median interval from initial diagnosis of melanoma to BM was 19.0 months (range 0.2–74.4 months). Most patients (68.0%) had more than 1 line of systemic treatment before BM.

**Table 1:** Baseline patient characteristics.

**Characteristic**	**Median (range) or *n* (%)**
Patient number	25 (100%)
Age	48y (25–77y)
Male	12 (48.0)
Female	13 (52.0)
**KPS score**	
70	1 (4.0)
80	8 (32.0)
90~100	16 (64.0)
**Primary tumor type**	
Cutaneous melanoma	8 (32.0)
Acral or mucosal melanoma	17 (68.0)
**BRAF V600 mutation**	
Yes	11 (44.0)
No	14 (56.0)
**C-kit mutation**	
Yes	2 (8.0)
No	10 (40.0)
Unknown	13 (52.0)
**LDH**	
<ULN	20 (80.0)
≥ULN	3 (12.0)
Unknown	2 (8.0)
**Interval between BM and initial diagnosis**	19.03 m (0.2–74.4 m)
**Lines of prior systemic treatment**	
0	8 (32.0)
1	6 (24.0)
2	5 (20.0)
≥3	6 (24.0)

*BM, Brain Metastasis; ULN, Upper Limit of Normal; LDH, Lactate Dehydrogenase*.

### BM Characteristics

Baseline BM characteristics are presented in Table 2. Most patients (60%) had more than one BM lesion. The median maximum diameter of BM was 10 mm (range, 2–51 mm). Extracranial metastasis was observed in 23 (92%) patients, 11 (44.0%) of them had more than 3 extracranial lesions. At the time of diagnosis, symptomatic BM was present in 7 (28.0%) patients, and 3 of them received steroids for symptom control. Two (8.0%) patients underwent surgical resection of BM before radiotherapy. Among all patients, 8 patients received ≥2 courses of radiotherapy for BM. Eighteen patients (72.0%) were treated with SBRT (stereotactic body radiation therapy) or SRS (stereotactic radiosurgery). Six (24.0%) patients received both SBRT/SRS and WBRT (whole brain radiation therapy), and one (4.0%) patient only received WBRT. Fourteen (56%) patients received radiotherapy before anti-PD-1 therapy. Eleven (44%) patients had started anti-PD-1 therapy before radiotherapy. All patients started receiving anti-PD-1 therapy in very recent years. Eighteen (72%) patients started receiving ICI therapy in 2018 and 2019, 5 (20%) patients in 2017, 2 (8%) patients in 2015. The median treatment period of anti-PD-1 monoclonal antibody was 10.77 months (range, 0.7–27.97). In the current study, we also calculated molGPA score which has been shown to be significantly correlated with survival of melanoma patients (24). The majority of patients (60%) had a molGPA score of 0–2.

**Table 2:** Baseline BM characteristics.

**Characteristic**	**Median (range) or *n* (%)**
**Total number of BM**	
1	10 (40.0)
2~4	8 (32.0)
5~10	4 (16.0)
>10	3 (12.0)
**Maximum diameter of BM**	10 mm (2–51 mm)
**Number of extracranial metastatic sites**	3 (0–7)
0	2 (8.0)
1~3	12 (48.0)
≥4	11 (44.0)
**BM with CNS symptoms**	
Yes	7 (28.0)
No	18 (72.0)
**Steroids for CNS symptoms**	
Yes	3 (12.0)
No	22 (88.0)
**Surgery for BM**	
Yes	2 (8.0)
No	23 (92.0)
**Radiotherapy for BM**	
SBRT or SRS	18 (72.0)
SBRT or SRS + WBRT	6 (24.0)
WBRT	1 (4.0)
**Treatment duration of anti-PD-1 therapy**	10.77 m (0.7–27.97 m)
**Melanoma-molGPA score**	2 (1–4)
0.0~2.0	15 (60.0)
2.5~4.0	10 (40.0)

*BM, Brain Metastasis; CNS, Central Nervous System; SBRT, Stereotactic Body Radiation Therapy; SRS, Stereotactic Radiosurgery; WBRT, Whole Brain Radiation Therapy; PD-1, Programmed Death 1; Melanoma-molGPA, Graded Prognostic Assessment for Melanoma Using Molecular Markers*.

### Treatment Efficacy

**Figure 2:**
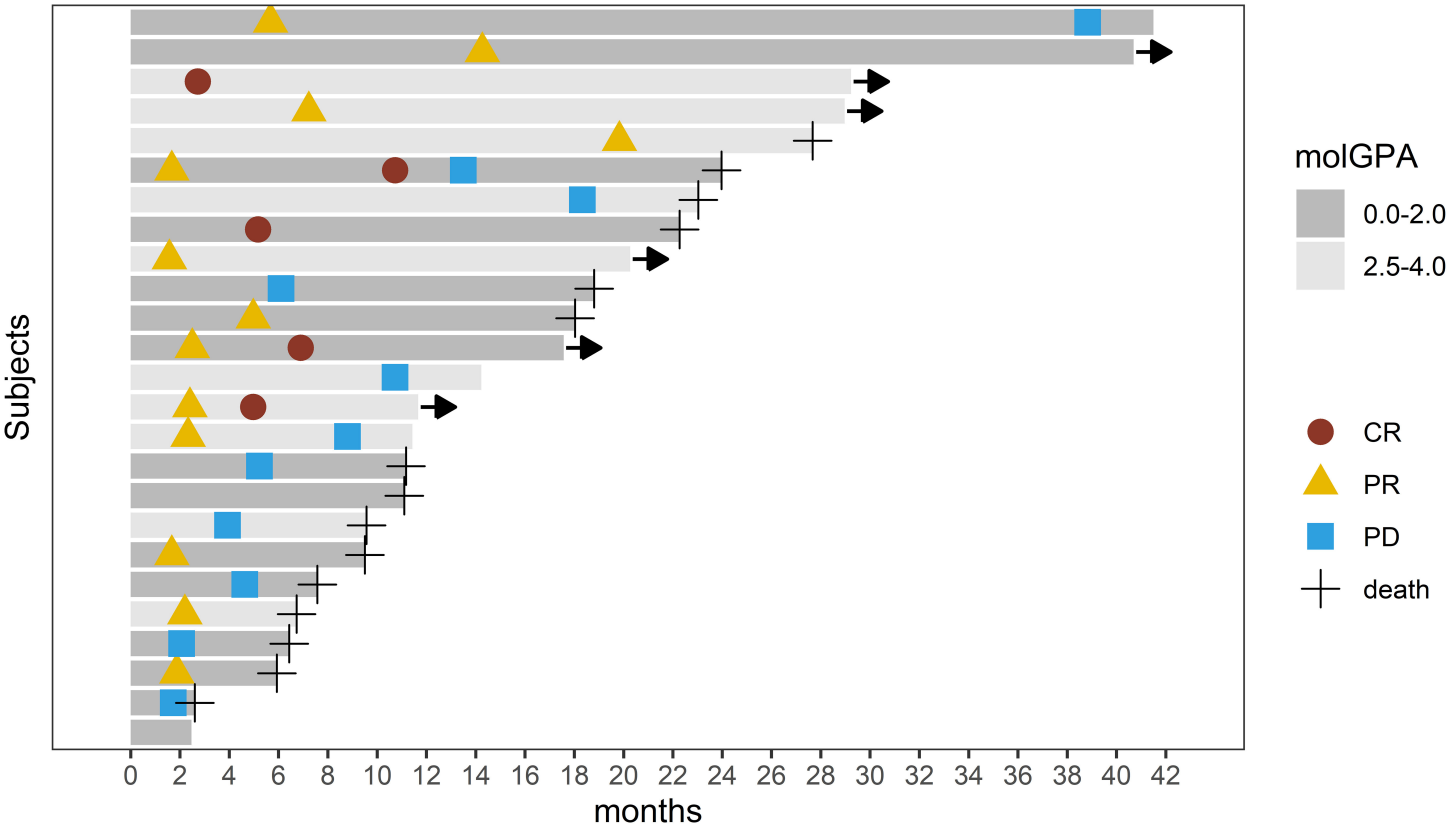
Swimmer's plot showing intracranial response and survival after patients with brain metastasis received radiotherapy and anti-PD-1 therapy. CR, Complete Response; PR, Partial Response; PD, Progression Disease. Arrow implies ongoing response.

**Figure 3:**
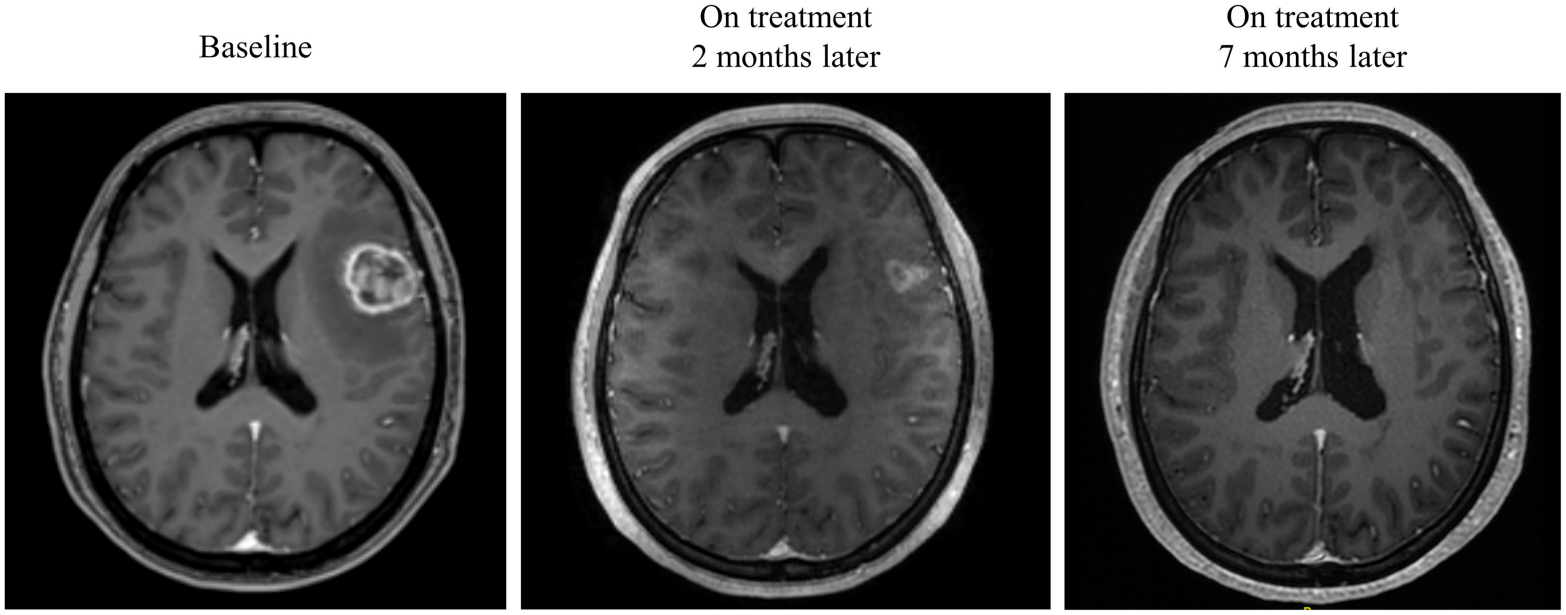
Radiological examples of intracranial complete response. A 40-year-old female diagnosed with melanoma BM was given anti-PD-1 therapy and radiotherapy. MRI images show a complete response 7 months later.

**Figure 4:**
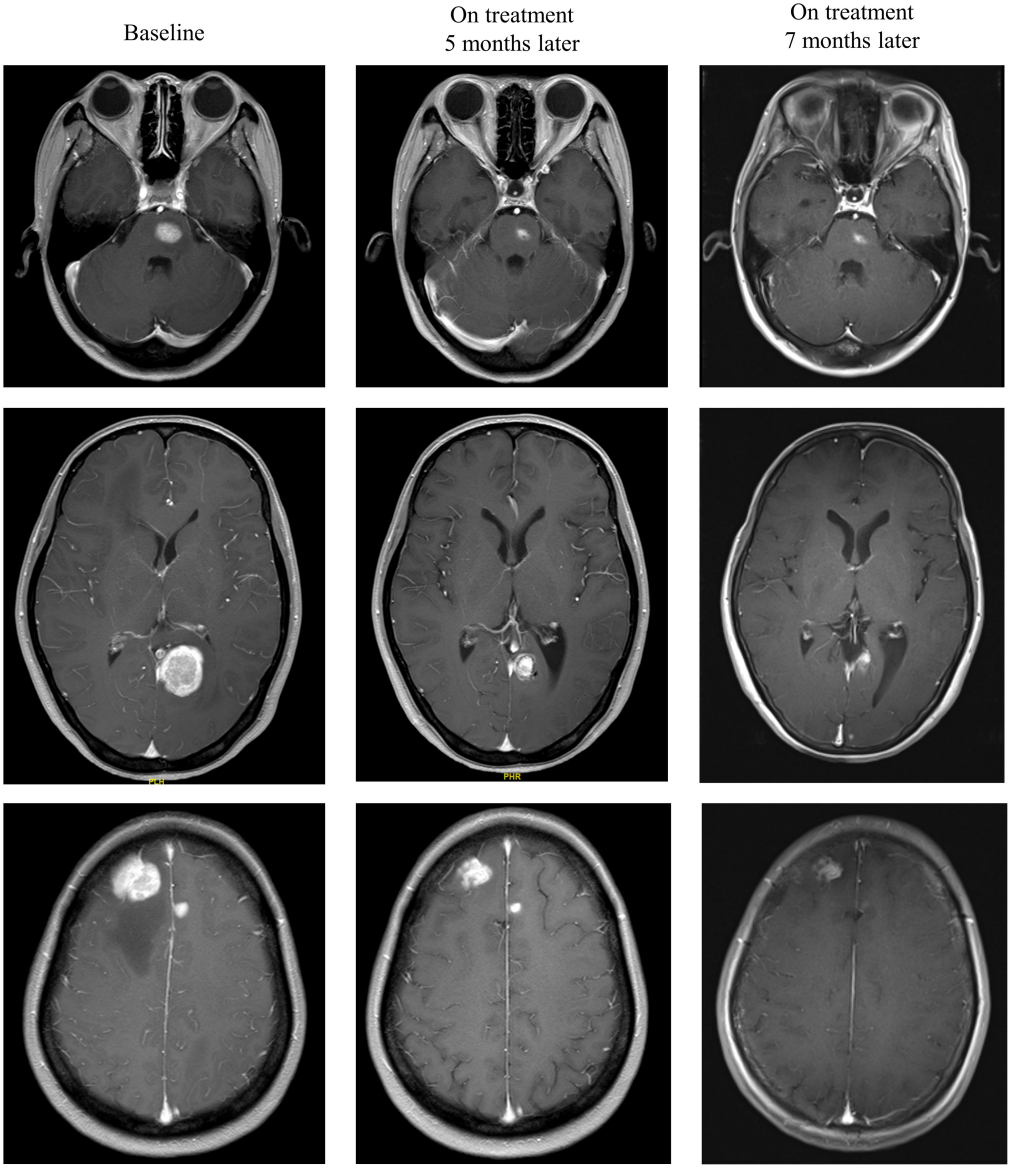
Radiological examples of intracranial partial response. A 33-year-old female diagnosed with multiple melanoma BMs was treated with anti-PD-1 therapy following radiotherapy. MRI images show a partial response 5 months later.

Intracranial response after patients treated with radiotherapy and anti-PD-1 therapy was shown in Figure 2. The objective intracranial response was observed in 15 (60%) patients with 5 (20%) patients achieving CR (complete response). And 6 (24%) patients showed an ongoing intracranial response at the time of data analysis. The MRI images of a CR-obtained case are shown in Figure 3. One female patient receiving anti-PD-1 therapy combined with radiotherapy achieved intracranial CR 7 months after diagnosis. Another patient diagnosed with multiple BMs was treated with anti-PD-1 therapy following radiotherapy had a partial response 5 months later (Figure 4).

**Figure 5:**
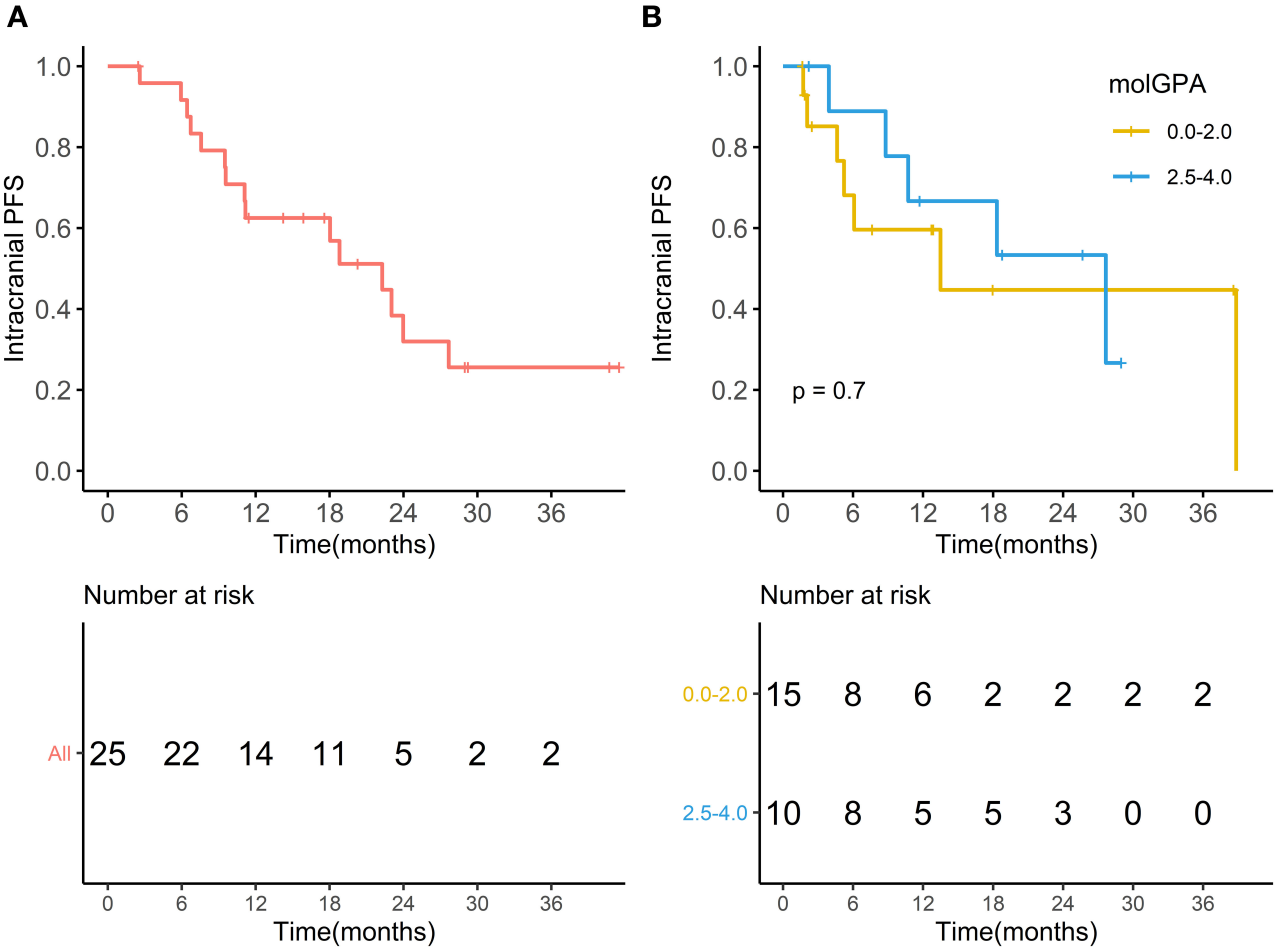
Intracranial progression free survival. **(A)** Intracranial progression free survival of all patients with BM treated with radiotherapy and anti-PD-1 therapy. **(B)** Intracranial progression free survival of patients with molGPA 0.0-2.0 vs. those with molGPA 2.5-4.0.

**Figure 6:**
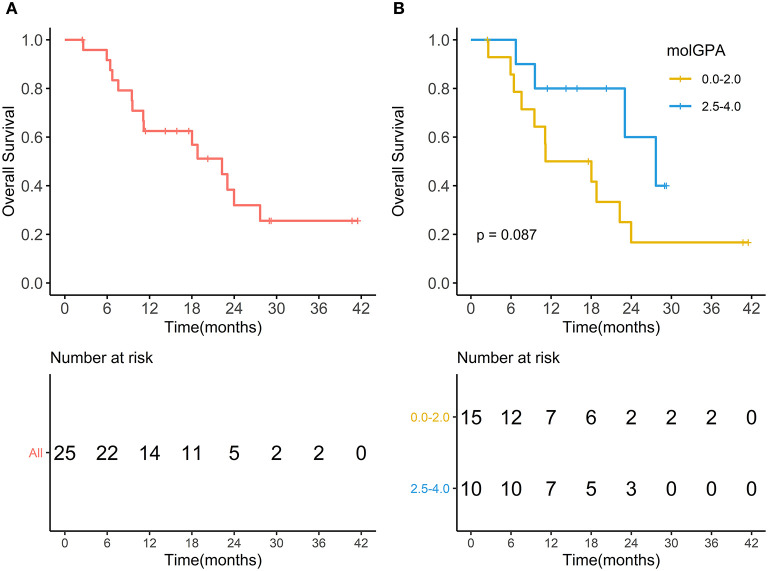
Overall survival. **(A)** Overall survival of all patients with brain metastases treated with radiotherapy and anti-PD-1 therapy. **(B)** Overall survival of patients with molGPA 0.0-2.0 and those with molGPA 2.5-4.0.

During the entire follow-up, 11 (44%) patients experienced intracranial PD (progressive disease), and 15 (60%) patients died. The median intracranial PFS was 10.73 months (range, 1.67–38.83 months), and the 1-year intracranial PFS was 61.9% (95% CI, 44.1–86.9%) (Figure 5A). The intracranial PFS was not significantly different between patients with melanoma-molGPA score of 0.0–2.0 and those with 2.5–4.0 (*p* = 0.7) (Figure 5B). The median OS was 15.87 months (range, 2.47–41.50 months), and the 1-year OS was 62.5% (95%CI, 45.8–85.2%) (Figure 6A). There was no statistically significant difference in OS between patients with a molGPA score of 0.0–2.0 and their counterparts of 2.5–4.0 (*p* = 0.087) (Figure 6B).

### Treatment-Related Adverse Events

The treatment-related AE (adverse events) were present in Table 3. There was no Grade 3 or higher treatment-related AE. The most frequently reported AE were rash (*n* = 10, 40.0%) and pruritus (*n* = 9, 36.0%). Other reported AE included skin hypopigmentation (*n* = 3, 12.0%), fatigue (*n* = 4, 16.0%), anorexia (*n* = 4, 16.0%), myalgia (*n* = 3, 12.0%), edema (*n* = 1, 4.0%), aminotransferase increased (*n* = 1, 4.0%), bilirubin increased (*n* = 1, 4.0%), tinnitus (*n* = 1, 4.0%), and hearing impaired (*n* = 1, 4.0%). Grade 2 immune-related psoriasis was reported by 1 patient (4.0%). Three patients (12.0%) had grade 1-2 hyperthyroidism or hypothyroidism. One patient developed grade 1 dysphasia after radiotherapy. Overall, no patient discontinued treatment due to AE.

**Table 3:** Treatment-related adverse events.

**CTCAE category**	**Grade 1**	**Grade 2**	**Grade 3–5**	**All (%)**
Rash	9	1	0	10 (40.0)
Pruritus	7	2	0	9 (36.0)
Skin hypopigmentation	3	0	0	3 (12.0)
Fatigue	3	1	0	4 (16.0)
Anorexia	4	0	0	4 (16.0)
Myalgia	3	0	0	3 (12.0)
Edema	1	0	0	1 (4.0)
Aminotransferase increased	0	1	0	1 (4.0)
Blood bilirubin increased	1	0	0	1 (4.0)
Tinnitus	1	0	0	1 (4.0)
Hearing impaired	1	0	0	1 (4.0)
Other skin disorder (psoriasis)	0	1	0	1 (4.0)
Hyperthyroidism or Hypothyroidism	2	1	0	3 (12.0)
Dysphasia	1	0	0	1 (4.0)

*CTCAE, Common Terminology Criteria for Adverse Events*.

## Discussion

Chinese melanoma patients show distinct clinical and molecular characteristics. Most Chinese patients were diagnosed with acral or mucosal melanoma which was believed to be associated with a worse prognosis (4, 6, 7). Compared with the Caucasian patients, T-cell inflammation, TMB (tumor mutational burden), and antigen presentation machinery of the Chinese patients are lower (5). MAPK (mitogen-activated protein kinase) pathway and TERT (telomerase reverse transcriptase) promoter gene mutations are also differentially represented in the Chinese population (4). However, few studies have reported the effectiveness of radiotherapy combined with systemic therapy in Chinese melanoma patients with BM, let alone radiotherapy combined with immunotherapy. Therefore, we systematically evaluate the efficacy of Chinese melanoma patients with BM treated with radiotherapy and anti-PD-1 therapy in the present study, and favorable outcomes are reported. We demonstrated that intracranial objective response was achieved in 60% patients. The median intracranial PFS and OS were 10.73 months (range, 1.67–38.83 months) and 15.87 months (range, 2.47–41.50 months), respectively. And the 1-year intracranial PFS, and OS were 61.9% (95% CI, 44.1–86.9%), and 62.5% (95%CI, 45.8–85.2%), respectively.

Generally, the prognosis of melanoma patients with BM is extremely poor. The median OS is only weeks for patients with untreated BM, and only 1.2–2.1 months for patients treated with BSC (best supportive care) (10, 12–14). When melanoma patients with BM were treated with local therapy combined with or without systemic therapy, their OS was slightly longer. Data from Royal Prince Alfred Hospital showed that the median OS of patients with BM receiving surgery and radiotherapy, surgery, radiotherapy was 8.9, 8.7, 3.4 months, respectively (12). A study from M. D. Anderson Cancer Center reported that the median OS of patients with BM undergoing surgery, SRS, chemotherapy, WBRT was 9.8, 7.7, 4.6, and 3.9 months, respectively (10). As targeted therapy using BRAF/MEK inhibitors has greatly improved the prognosis of patients with metastatic melanoma, the efficacy of these inhibitors has also been explored in patients with BM. When patients were treated with dabrafenib for progressive BM after previous local treatments, the median OS was 7.33 months (16). The median OS for patients receiving vemurafenib with or without local treatments (radiotherapy or surgery) was 8.9–9.6 months (17).

More recently, immunotherapy has shown impressive and favorable responses in melanoma patients with BM. A study evaluated the efficacy of 18 melanoma patients treated with PD-1 inhibitor pembrolizumab, 4 (22%) patients achieved brain metastasis response (18). In another study, objective response was observed in 46–57% of patients who were treated with nivolumab and ipilimumab (19, 20). Median OS of 9.9 months was reported in patients receiving anti-PD-1 therapy with or without local therapy at five major melanoma centers in Australia (21). Preclinical and clinical data have demonstrated the synergistic anti-tumor effect of immunotherapy and radiotherapy (25–30). Data from Memorial Sloan-Kettering Cancer Center indicated that the median OS of patients receiving ipilimumab and SRS was 12.4 months (27). In another study, the median OS of patients who were treated with ipilimumab and radiotherapy in Brigham and Women's Hospital and Dana-Farber Cancer Institute was 14 months (28). In the current study, intracranial objective response was observed in 60% patients, and a median OS of 15.87 months was reported in patients treated with anti-PD-1 therapy and radiotherapy. Moreover, the patients in our study developed brain metastases earlier. The median time from initial diagnosis of melanoma to cerebral metastasis was 2.7 years in the U.S, and 3.1 years in Australia (11). In the present study, the median time from primary diagnosis of melanoma to BM was 19 months. This suggests that Chinese melanoma patients with BM may benefit more from the combination of anti-PD-1 therapy and radiotherapy.

Several studies have compared the outcome of patients with BM receiving different types of systemic therapies following radiotherapy. At Melanoma Institute Australia, the median OS of patients receiving anti-CTLA4, anti-PD-1, BRAFi ± MEKi, and no systemic drug therapy at the time of SRS was 7.5, 20.4, 17.8, and 10.8 months, respectively. However, the statistical analysis results were not reported (30). Another study from H. Lee Moffitt Cancer Center and Research Institute showed that 12-month OS rates of anti-PD-1 therapy, anti-CTLA-4 therapy, BRAF/MEKi, BRAFi, and chemotherapy following SRS were 48, 41, 65, 24, and 10%, respectively (*p* = 0.01) (29). It seems that patients treated with targeted therapy following SRS had a better OS. Prospective randomized clinical trials are required for further verification. And more data about the efficacy of systemic therapies and radiotherapy in Chinese melanoma patients with BM are demanded to guide individualized therapy.

There are some limitations to this study. First, this study is retrospective. And the data analyzed was from single center. Although we recorded patient survival data as accurately as possible, the data were not collected in a standardized and prospective manner. Further prospective and multi-centers clinical trials are required to demonstrate the efficacy of the combination of anti-PD-1 therapy and radiotherapy in treating melanoma BM. Secondly, since anti-PD-1 therapy has been used to treat Chinese melanoma patients in very recent years, the sample size of the present study is relatively small. In this study, we analyzed the value of melanoma-molGPA score in predicting intracranial PFS and OS for patients with melanoma BM. Perhaps due to the small sample size, we cannot find the significant difference in intracranial PFS and OS between patients with high and low molGPA scores. Larger sample size is warranted to further verify the results.

In conclusion, the median OS of patients with melanoma BM who received both radiotherapy and anti-PD-1 therapy was 15.87 months in the present study. It provides evidence of combining anti-PD-1 therapy and radiotherapy in the management of melanoma BM. However, differences in patient-related factors may affect the outcome, and formal randomized clinical trials are required to determine whether anti-PD-1 therapy and radiotherapy have a synergistic anti-tumor effect in the setting of melanoma BM. Current ongoing clinical trials will provide further prospective evidence. A pilot study (NCT02716948) is investigating the side effects of SRS and nivolumab in treating patients with melanoma metastases in the brain or spine. Another pilot study (NCT02858869) is also exploring the adverse effects of pembrolizumab and SRS for Melanoma BM. The randomized ABC-X trial (NCT03340129) is evaluating the efficacy of ipilimumab and nivolumab with or without concurrent SRS in patients with asymptomatic, untreated melanoma BM. As the treatment paradigm for patients with melanoma BM evolves, choosing the appropriate systemic treatment or combination therapy and the optimal sequence of systemic and local therapies will be the next challenge for oncologists (21).

## Data Availability Statement

The original contributions presented in the study are included in the article/supplementary material, further inquiries can be directed to the corresponding author/s.

## Ethics Statement

The studies involving human participants were reviewed and approved by Institutional Review Board of Sun Yat-Sen University Cancer Center. Written informed consent for participation was not required for this study in accordance with the national legislation and the institutional requirements.

## Author Contributions

ZWP and LXL designed the study. LXL contributed to patients treatment and management. SW, CPY, and LLG collected and analyzed the data. SW wrote the manuscript. YC and LC provided suggestions for manuscript writing and data analysis. All authors reviewed and approved this manuscript.

## Conflict of Interest

The authors declare that the research was conducted in the absence of any commercial or financial relationships that could be construed as a potential conflict of interest.
